# High performance ozone based advanced oxidation processes catalyzed with novel argon plasma treated iron oxyhydroxide hydrate for phenazopyridine degradation

**DOI:** 10.1038/s41598-020-80200-9

**Published:** 2021-01-13

**Authors:** Rasool Pelalak, Zahra Heidari, Mojtaba Forouzesh, Eslam Ghareshabani, Reza Alizadeh, Azam Marjani, Saeed Shirazian

**Affiliations:** 1 Institute of Research and Development, Duy Tan University, Da Nang, 550000 Vietnam; 2 Faculty of Environmental and Chemical Engineering, Duy Tan University, Da Nang, 550000 Vietnam; 3 Chemical Engineering Faculty, Sahand University of Technology, Sahand New Town, Tabriz, 51335-1996 Iran; 4 Physics Faculty, Sahand University of Technology, Sahand New Town, Tabriz, 51335-1996 Iran; 5 Department for Management of Science and Technology Development, Ton Duc Thang University, Ho Chi Minh City, Vietnam; 6 Faculty of Applied Sciences, Ton Duc Thang University, Ho Chi Minh City, Vietnam; 7 Laboratory of Computational Modeling of Drugs, South Ural State University, 76 Lenin prospekt, Chelyabinsk, Russia 454080

**Keywords:** Energy science and technology, Engineering, Nanoscience and technology, Physics

## Abstract

The present study has focused on the degradation of phenazopyridine (PhP) as an emerging contaminant through catalytic ozonation by novel plasma treated natural limonite (FeOOH·xH_2_O, NL) under argon atmosphere (PTL/Ar). The physical and chemical characteristics of samples were evaluated with different analyses. The obtained results demonstrated higher surface area for PTL/Ar and negligible change in crystal structure, compared to NL. It was found that the synergistic effect between ozone and PTL/Ar nanocatalyst was led to highest PhP degradation efficiency. The kinetic study confirmed the pseudo-first-order reaction for the PhP degradation processes included adsorption, peroxone and ozonation, catalytic ozonation with NL and PTL/Ar. Long term application (6 cycles) confirmed the high stability of the PTL/Ar. Moreover, different organic and inorganic salts as well as the dissolved ozone concentration demonstrated the predominant role of hydroxyl radicals and superoxide radicals in PhP degradation by catalytic Ozonation using PTL/Ar. The main produced intermediates during PhP oxidation by PTL/Ar catalytic ozonation were identified using LC–(+ESI)–MS technique. Finally, the negligible iron leaching, higher mineralization rate, lower electrical energy consumption and excellent catalytic activity of PTL/Ar samples demonstrate the superior application of non-thermal plasma for treatment of NL.

## Introduction

The pharmaceutical wastewaters contain extensive amounts of bio-resistant contaminants that are not effectively remediated through conventional biological methods such as activated sludge, sequencing batch reactor, biofilm reactors, etc.^1,2^. Furthermore, physical processes like adsorption, coagulation or membrane processes are not favorable because pollutants only transfer from one phase to another that needs more treatment^3–6^. In the recent decay, advanced oxidation processes (AOPs) are of great interest due to their potential to degrade any resistant contaminants by in-situ generation of active oxidizing agents, good mineralization of pollutants and no extra waste^7–9^. Among different types of AOPs, much attention has been paid to the ozone based processes because of its accessibility, feasibility, high reactivity and purification of final effluent^10–12^. Ozone with 2.07 V redox potential destroys organic contaminants through direct oxidation of pollutants and/or indirect mechanisms by generation free hydroxyl radical ($^{ \cdot } OH$). Despite many advantages, the ozonation process was restricted by some drawbacks such as solubility limitation and high cost of ozone generation^13^. To overcome these limitation, combined methods are a promising solution which increases the efficiency of process by creating a synergistic effect, producing more reactive species, increasing the mineralization rate and reducing the reaction time^11^. In recent years, catalytic ozonation process is one of the most promising AOPs. In this process, the decomposition of ozone is enhanced by applying a catalyst, leading to formation powerful hydroxyl radicals and other radical species, which is capable to oxidize the most of refractory compounds to more biodegradable and less toxic intermediates^14^. Both heterogeneous and homogenous catalytic ozonation processes were applied; however, the heterogeneous processes is more favorable because of the simplicity of process, ease of catalyst separation and no secondary pollution^15^. Nevertheless, mass transfer resistance due to the particle size and few reaction sites are the main drawbacks of heterogeneous operation which can be solved by using nanostructured materials^16^. Numerous catalysts have been used in the literature including metal oxides, activated carbon, natural and synthetic materials^17–21^. The low-cost and abundant natural iron oxides are available in form of crystalline minerals such as goethite, ilmenite, limonite, magnetite and hematite^18^. Limonite is one of these minerals that is widely distributed around the globe. Natura limonite (NL) contains fine crystal grains from multiphase mixture with porous structure and large specific surface area as well as a high surface energy which results in a stronger capability. The salient property of NL that enhances its catalytic activity is the presence of superficial hydroxyl groups on its surface which participates in the production of hydroxyl radicals^22^. The modification of natural minerals is an effective way to increase their application, which is done using both physical and/or chemical processes. Mechanical modification is a simple, inexpensive and efficient method to reduce the size of minerals from micrometers to nanometers. Furthermore, chemical activation is effective to enhance surface hydroxyl groups as well as specific surface area^18,23^. From different types of modification methods, non-thermal plasma technique is one of the newest methods for surface modification. The main feature of plasma technology is environmental-friendly, cost-effective, quickness, simplicity and ability of various nanostructures production^24^. Phenazopyridine hydrochloride (PhP) is a heterocyclic aromatic azo-compound that is used to help with the irritation, pain or urgency caused by urinary tract infections^25^. Due to the low water solubility (0.202 g/L) and low biodegradability, PhP accumulates in the sewages and therefore, treatment of PhP contaminated waters are very important^26^.

To the best of our knowledge, there is no comprehensive research on non-thermal glow discharge plasma method for treatment of NL in order to improve the structural properties and catalytic activity of this iron mineral. Different characterization analysis such as: FESEM, XRD, AAS, TEM, FTIR, BET-BJH, pH_pzc_ and EDX confirmed the physical and chemical characteristics of synthesized samples. Moreover, in this research, the influence of the eco-friendly modified NL was evaluated by degradation of PhP antibiotic through catalytic ozonation process. For this purpose, the effect of main process parameters such as catalyst dosage, solution pH, initial contaminant and ozone concentration were investigated on the performance of process. In the following sections, the catalyst stability and reusability of as-prepared catalysts, Fe ions release, mineralization and also assessment of electrical energy consumption were also investigated. By evaluating the effect of organic and inorganic salts and also, the dissolved ozone concentration the PhP degradation mechanism was proposed. In addition the LC–( ESI)–MS/MS analysis used to recognize the produced intermediates and by-products during the PhP degradation by catalytic ozonation using PTL/Ar nanocatalysts. Only few reports have been worked on PhP oxidation intermediates in ozone based AOPs.

## Materials and methods

### Materials

NL ore was provided from Tekab iron mine (Est Azerbaijan, Iran). The PhP was used as pharmaceutical contaminant which characteristics are given in Table 1. Sodium thiosulfate (Na_2_S_2_O_3_.5H_2_O) as ozonation reaction quenching, sodium hydroxide (NaOH) and sulfuric acid (H_2_SO_4_) for pH adjustment, potassium hydrogen phthalate (C_8_H_5_KO_4_, purity of 99.5%) for TOC calibration and, scavenging agents such as t-butanol (C_4_H_10_O), benzoquinone (BQ), sodium dihydrogen phosphate (NaH_2_PO_4_), sodium carbonate (Na_2_CO_3_) and sodium nitrate (NaNO_3_) were provided from Merck Co. (Germany). Ultrapure water used for all experimental tests. All reagent used in the AOPs were pure and used as received without any purification.

**Table 1:** Characteristics of phenazopyridine hydrochlorid.

Molecular structure	C_11_H_11_N_5_·HCl
Molecular weight (g/mol)	249.7
λ_max_ (nm)	430
Melting point (°C)	139
pK_a_	5.05
Water solubility (g/L)	0.202

### Preparation of limonite nanostructures

**Figure 1:**
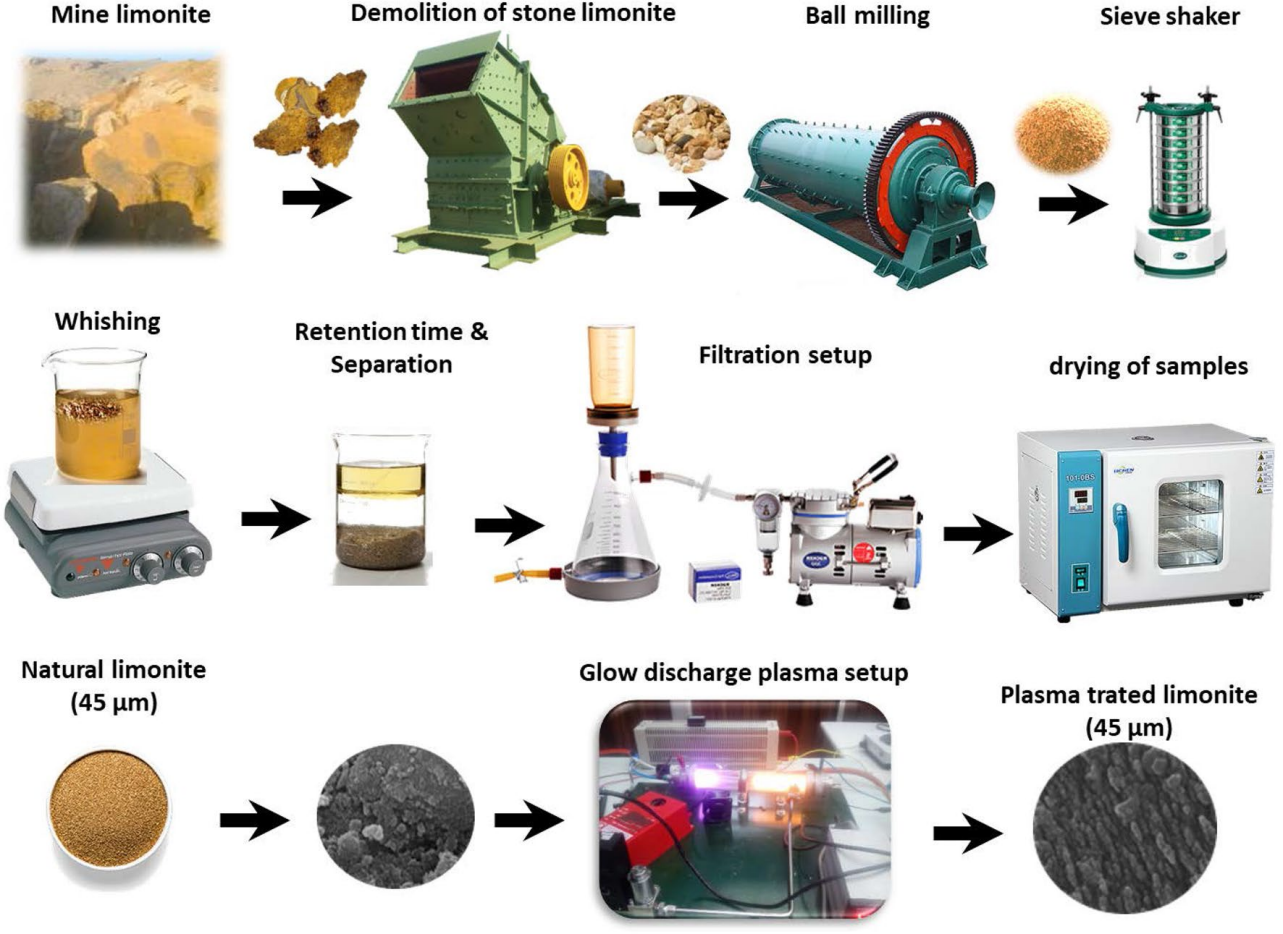
Graphic setup of the plasma treatment system for producing PTL/Ar nanostructures.

The limonite nanostructures were produced from NL by non-thermal glow discharge plasma under argon atmosphere. The treatment procedure is schematically presented in Fig. 1. At first, NL samples were crushed by rod and ball milling to obtain microstructure particles. After sieving, washing with ultrapure water, filtration and drying at 70 °C, 3 g of the limonite was placed in a Pyrex tube plasma reactor, which was completely sealed with aluminum bonnets on both sides^12^. High-voltage direct current (DC) supplied by a DC power source was connected by the aluminum bonnets to generate glow discharge plasma. Argon gas with flowrate of 3 cm^3^/s was pumped within the reactor to bring the pressure to 50 Pa. The NL plasma-treated under Ar was denoted as PTL/Ar.

### Characterizations of limonites

The crystalline structure of NL and PTL/Ar samples were evaluated by X-ray diffraction (XRD) (Siemens D5000 diffractometer, 2θ = 10–80) armed with Cu-Kα (45 kV, 40 mA, λ = 1.54178 Å). The size and morphology of the NL and PTL/Ar samples were investigated using Field Emission.

Scanning Electron Microscopy equipped with an EDX microanalysis (FESEM; DSM-960A Zeiss, Germany) and Transmission Electron Microscopy (TEM; LEO 912AB). Fourier Transform Infrared spectra (FTIR) were conducted in Bruker Tensor 27 (400–4000 1/cm). The specific surface area (S_BET_), porosity, pore diameter and volume of NL and PTL/Ar samples were calculated by physical nitrogen adsorption and desorption experiment (at 77 K and room temperature, respectively) using Brunauer Emmett Teller (BET) analysis (ChemBET3000, USA). The Microstructure Distance Measurement software was used to evaluate particle size distribution of the nanoparticles (Nahamin Pardazan Asia Co., Iran). The atomic absorption spectroscopy (AAS) analyses measured the concentration of Fe ions released in the aquatic medium (Novaa 400 Analytikjena). The point of zero charge (pH_PZC_) was performed by a procedure proposed by Mustafa et al.^27^. In this method, ten glass bottles containing 40 mL NaNO_3_ (0.1 M) in ultrapure water with different pH values between 2 and 11 were prepared, each bottles containing 0.2 g of NL or PTL/Ar. The suspensions stirred for 48 h at 25 ℃. The pH_PZC_ is defined by plotting the final pH versus initial pH.

### Ozone based advanced oxidation processes experiments

**Figure 2:**
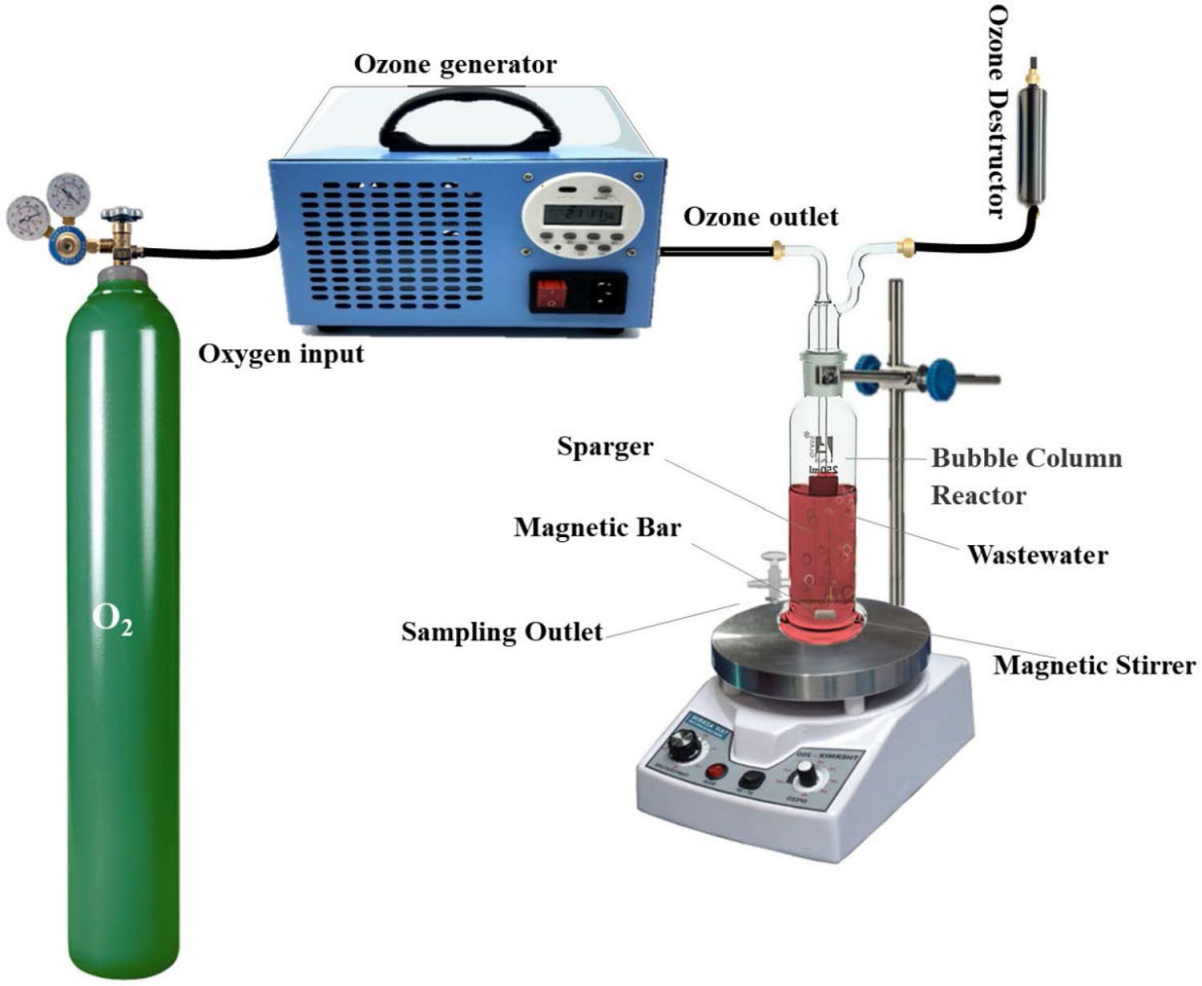
Schematic diagram of catalytic ozonation process.

Semi-batch experiments of ozonation based processes were performed in a tubular Pyrex reactor with 4 cm internal diameter and 35 cm in height, as illustrated in Fig. 2. Ozone was generated using Ozomatic ozone generator (Germany) and through a diffuser entered to the reaction medium. In each experiment, 250 mL solution was prepared containing desirable concentration of PhP between 30 and 60 mg/L and, catalyst dosage between 0.25 and 1 g/L in deionized water. Afterward, the solution was treated by chosen different ozone concentration between 7 and 15 mg/L. At different time of reactions samples were taken from the reactor and quenched immediately with Na_2_S_2_O_3_ (0.01 M). Then, filtered by a 0.45 µm PTFE filter for further analysis.

### Analytical methods

The concentration of PhP at different times of reaction was evaluated at 270 nm through a UV–Vis spectrophotometer (WPA Lightwave S2000, UK). A VCSH TOC analyzer (Shimadzu, Japan) was conducted to measure the Total organic carbon (TOC) of samples. The residual ozone concentration in the solution was evaluated by Indigo colorimetric procedure^28^.

The degradation percent of PhP and TOC removal were calculated using Eq. (1) where M_0_ (mg/L) and M_t_ (mg/L) are the concentration/TOC at initial and t (min) time of concentration, respectively.

1 $$ {\text{M}}\,{\text{degradation}}\,{\text{or}}\,{\text{removal}}\,\left( {\text{\% }} \right) = \frac{{{\text{M}}_{0} - {\text{M}}_{{\text{t}}} }}{{{\text{M}}_{0} }} \times 100\quad { }\left( {{\text{M}}\,{\text{represented }}\,{\text{PhP}}\,{\text{or }}\,{\text{TOC}}} \right) $$

The kinetic rates of reaction were evaluated using pseudo first-order kinetics (Eq. (2)):

2 $$ {\text{ln}}\left( {\frac{{C_{0} }}{C}} \right) = {\text{kt}} $$

where C_0_ and C (mg/L) are the initial and final concentration of PhP, respectively. The time of reaction and the apparent first-order rate constant are represented by k (1/min) and t (min), respectively.

### LC–MS/MS analysis

The liquid chromatography–mass spectrometry (LC-(+ ESI)-MS) technology was utilized to monitor the produced intermediates and by-products and propose the PhP degradation pathways in catalytic ozonation by PTL/Ar. The LC–MS/MS analyses were performed using mass spectrum (EMS) scan with positive mode on a 3200 QTrap mass spectrometer (AB SCIEX, Framingham, USA). The measurements were carried out at 254 nm in a C18 column (100 Å, 100 mm × 2.1 mm). Pure acetonitrile (CH_3_CN, LC–MS grade) and H_2_O (30:70) were used as the mobile phase. The LC–MS/MS was scanned in the mass region from 50 to 1000 m/z with sampling period of 0.1 s. The CDL and heating module temperature were both set as 350 °C and 450 °C, respectively.

## Results and discussion

### Physical and chemical characterizations of limonite

#### XRD

**Figure 3:**
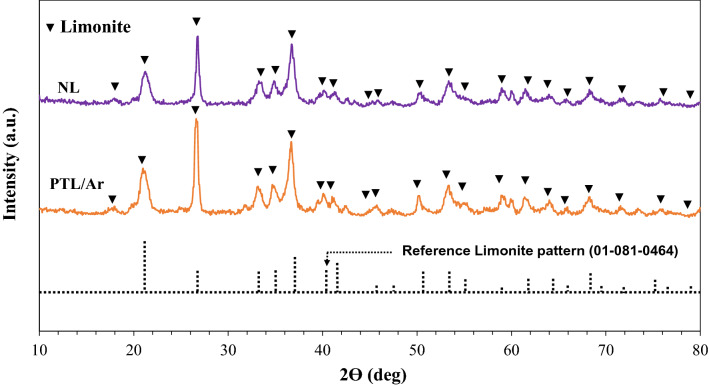
The XRD spectra of NL and PTL/Ar.

XRD patterns of the limonite before and after plasma treatment are presented in Fig. 3. diffraction peaks at 2θ values of 17.79°, 21.24°, 26.34°, 33.24°, 34.68°, 47.25°, 50.63°, 53.24°, and 68.50°, which were associated to the characteristics (020), (011), (021), (031), (120), (111), (112), (122) and (103) plans. The observations in the XRD patterns of the natural and plasma-treated samples indicated insignificant changes in the position of the XRD peaks after plasma treatment.

#### FESEM

**Figure 4:**
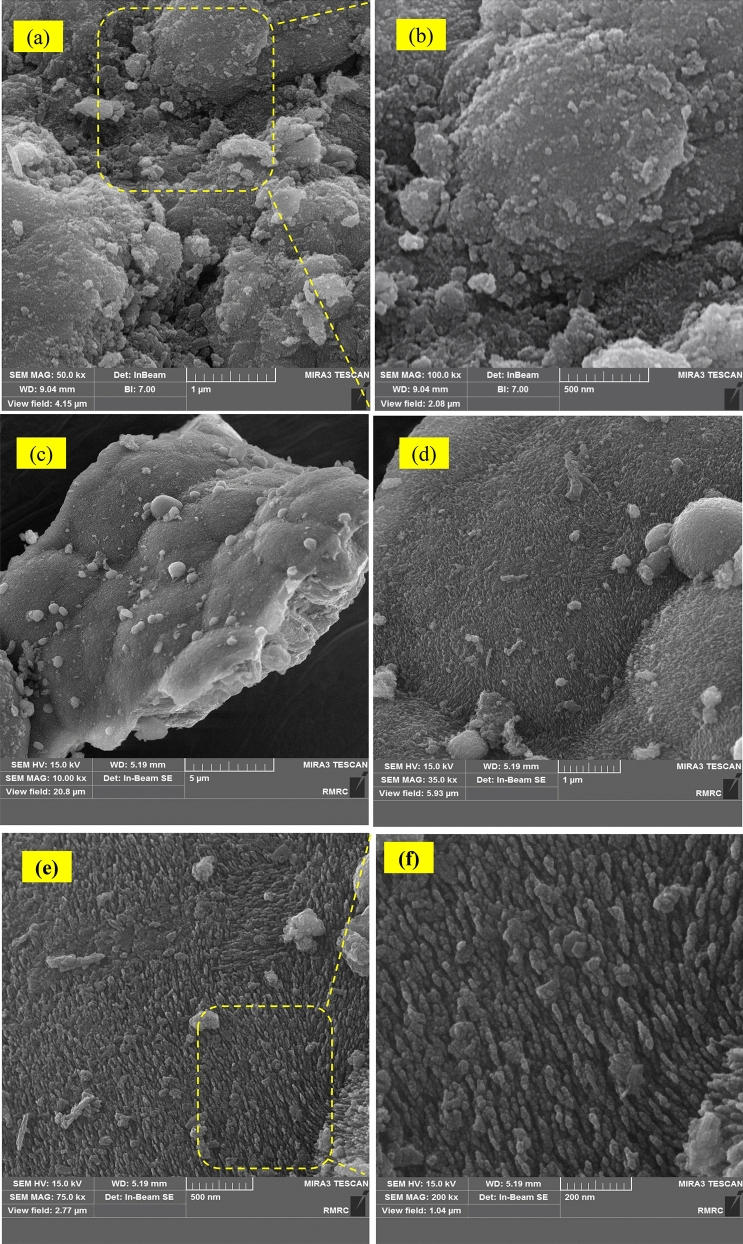
FESEM micrographs of (**a**, **b**) NL samples and (**c**–**f**) PTL/Ar samples.

FESEM analysis was conducted to investigate the surface morphology of samples. The FESEM images of NL and PTL/Ar samples with different magnifications are presented in Fig. 4. The bulky structure with various morphologies was observed in the NL samples (Fig. 4a,b) while smaller size with uniform morphology of PTL/Ar samples can be clearly seen in Fig. 4c–f. Plasma environment resulted in bombardment of the nanocatalyst surface, provided more breakups in particles and caused a homogenous distribution. The high-energy of argon molecules had an important effect in the treatment procedure and provided smoother nanocatalyst particles and larger contacting interfaces of α-FeOOH nanocatalysts consequently, the mass transport limitations could be lower compared to non-treated samples. As the results indicated an improvement in morphological characteristics of limonite samples was observed after plasma-treatment. Moreover, it can be said the smaller particle size, higher porosity and the rough structure of plasma treated samples will increase the catalytic activity of PTL/Ar.

#### EDX, TEM and particle size distribution

**Figure 5:**
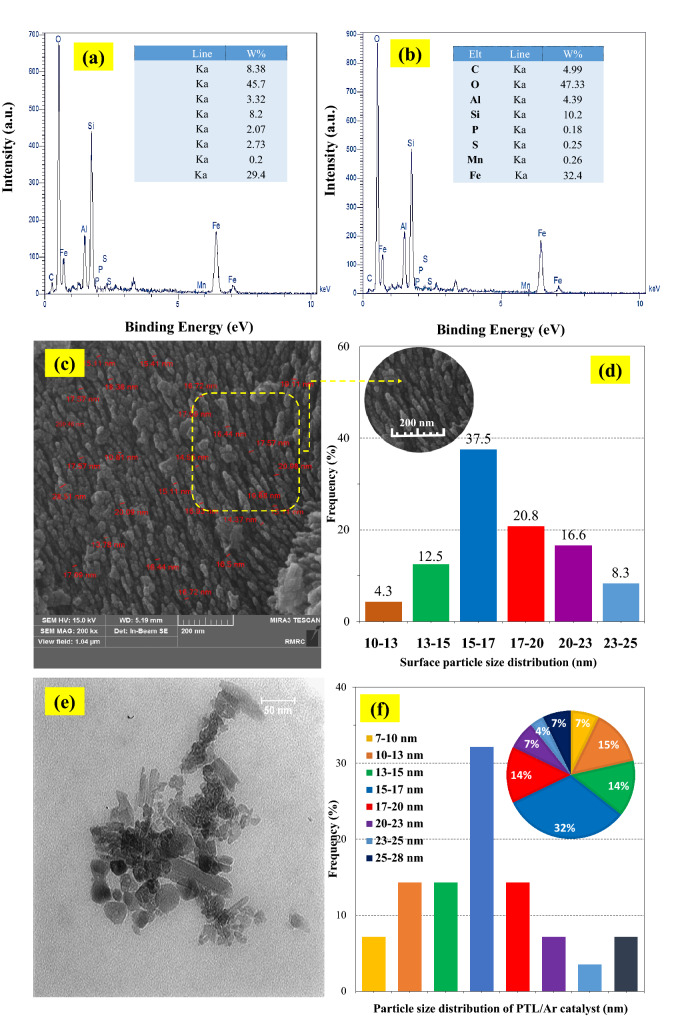
EDX and elemental analysis of (**a**) NL and (**b**) PTL/Ar; particle size distribution of PTL/Ar according FESEM (**c**, **d**) and TEM (**e**, **f**) analysis.

The results of EDX analysis for the samples before and after plasma treatment were shown in Fig. 5a,b, respectively. The inset tables of these graphs showed the presence of Fe, O, C and Si in higher amounts which indicated the purity of the precursors. Moreover, these results proved the presence of elements were not changed before and after plasma treatment. On the other hand, the plasma treatment increased the Fe atoms in the surface of nanocatalysts. It can be clearly seen that by increasing the intensity of the Fe peaks the intensity of C peak in the treated samples were decreased. As a consequence of this reduction the surface of treated samples made free of carbon impurities. Further studies on PTL/Ar surface morphology were performed by TEM analysis and the micrograph was shown in Fig. 5e. For detailed analysis, Fig. 5c,d,f illustrated the diameter size distribution of particles on the surface of PTL/Ar derived from FESEM and TEM micrographs. The as prepared nanocatalyst has a narrow size distribution. Accordingly, all particles were less than 28 nm in diameter size. Also, the major portion of the particles is in the range of 15–17 nm (37.5%).

**Figure 6:**
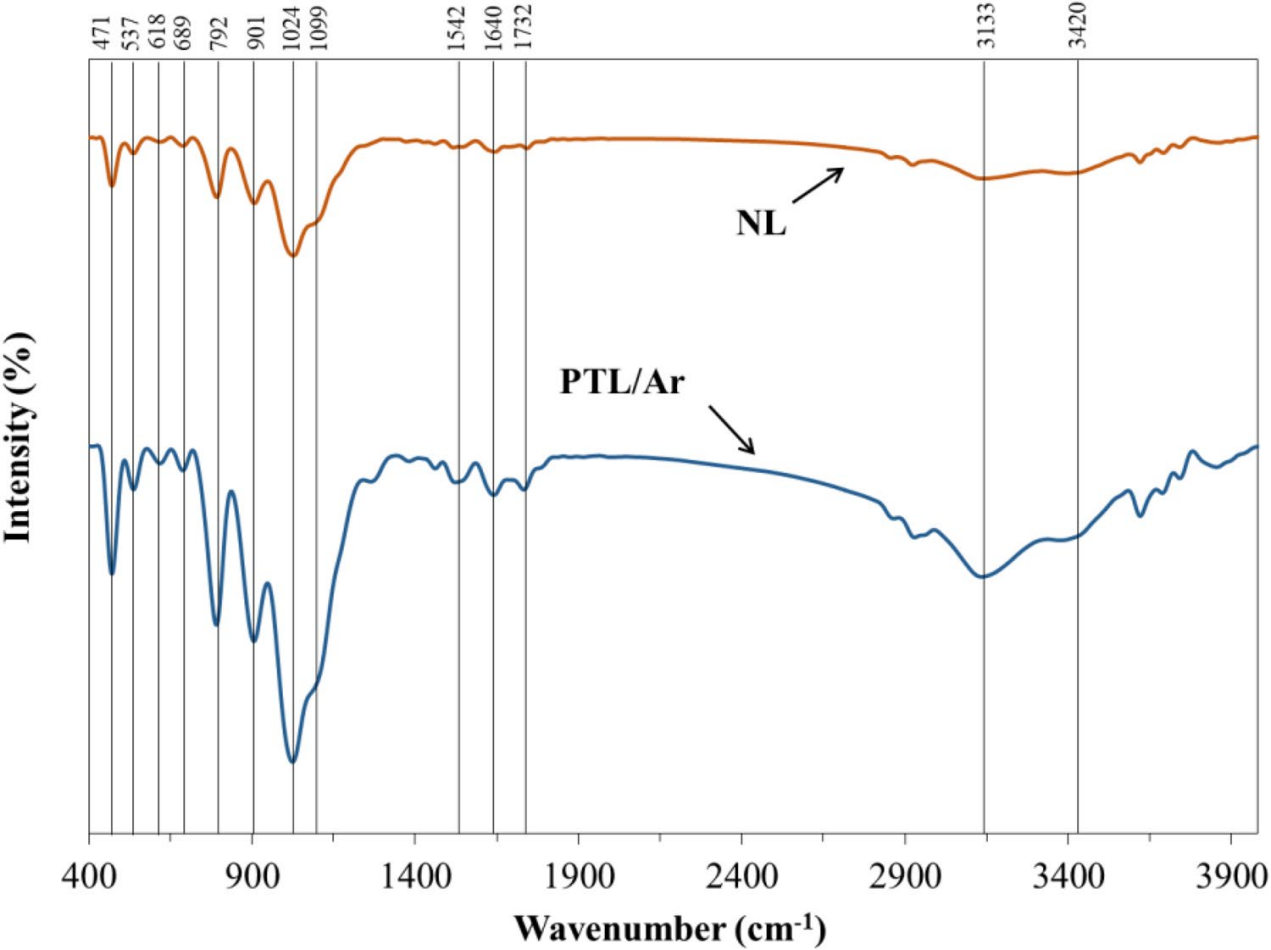
The FT-IR spectra of NL and PTL/Ar.

#### FTIR

The FT-IR spectra of both NL and PTL/Ar samples in the range of 400–4000 cm^−1^ are shown in Fig. 6. In both natural and treated samples, adsorption bonds around 1640 and 3420 cm^−1^ can be related to O–H bond in water molecules^29^. Also, the peak around 3650 cm^−1^ is assigned to OH-groups^30^. The peaks in the range of 1542 and 1640 cm^−1^ can be related to vibration mode of the C–H bond^29,31^. The peaks in 1732 cm^−1^ region are associated with the C=O vibrations. The peaks at about 537 and 471 cm^−1^ may be related to the tensile vibrations of the Fe–O bond. Furthermore, the absorption bands at around 792, 901 and 1024 cm^−1^ are also related to the stretching mode of the surface Fe-OH groups, which are the most important factor in the formation of surface hydroxyl groups^12^. A significant increase in the intensity of Fe-related peaks is observed in the plasma-treated sample. This phenomenon can be related to the increase in purity as well as the increase in surface iron after plasma processing. In addition, the vibrations at 792, 901, and 1024 cm^−1^ also related to Fe-OH surface groups which significantly increased by plasma treatment. Also, the location of the available peaks is similar for both samples which confirm that the surface functional groups are the same for NL and PTL/Ar samples.

#### Textural properties

**Figure 7:**
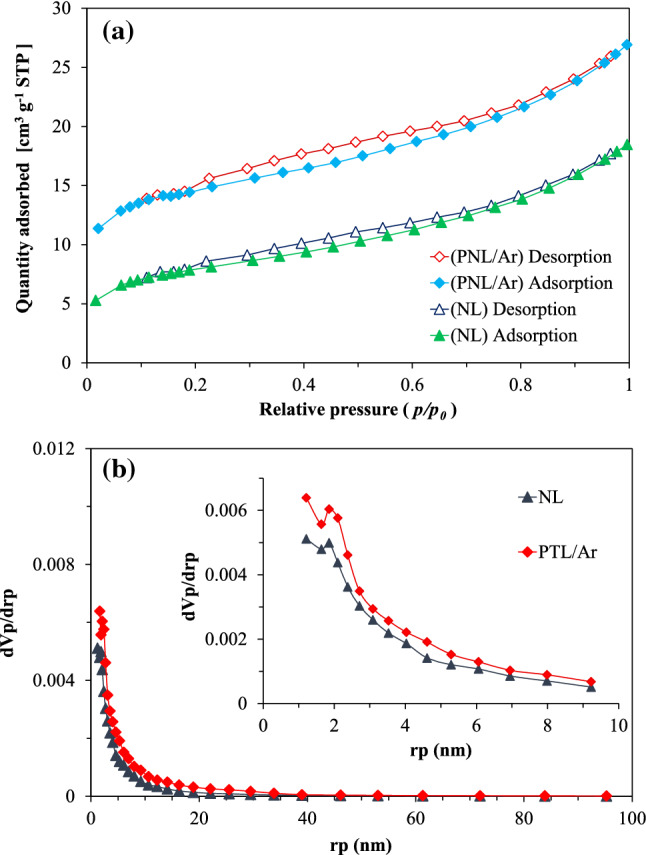
The nitrogen adsorption/desorption isotherms (**a**) and pore size distribution for NL and PTL/Ar (**b**).

BET-BJH analysis was performed for determination of the specific surface area, pore diameter and volume of the raw and treated samples. Figure 7a,b, presented the pore size distribution and N_2_ adsorption–desorption isotherms for NL and PTL/Ar. As it is obvious both samples showed Type-IV isotherm behavior (ISO 15,901–2) with a hysteresis cycle, revealing the porous structure with meso–micro cavities. The obtained results also indicated that the closed pores converted to interconnected open cavities. Also, the H4 type hysteresis can be considered for limonite samples, indicating narrow gap cavities. The textural properties of NL and PTL/Ar including total pore volume, pore diameter and specific surface area are listed in Table 2. From Ar adsorption/desorption experiment, limonite has a surface area of 33.37 m^2^/g which increased to 49.16 m^2^/g after plasma treatment. The pore volume of PTL/Ar samples was obtained about 0.039 cm^3^/g which improved significantly compared to NL samples (0.023 cm^3^/g) as a consequence of plasma treatment. This increase in specific surface area results in the generation of more active sites on the surface of the catalyst and consequently, enhances the performance of catalytic process.

**Table 2:** Structural properties of NL and PTL/Ar.

Sample	Pore diameter (nm)	Pore volume (cm^3^/g)	S_BET_ (m^2^/g)
NL	4.83	0.023	33.37
PTL/Ar	3.1	0.039	49.16

#### pH_PZC_ determination

**Figure 8:**
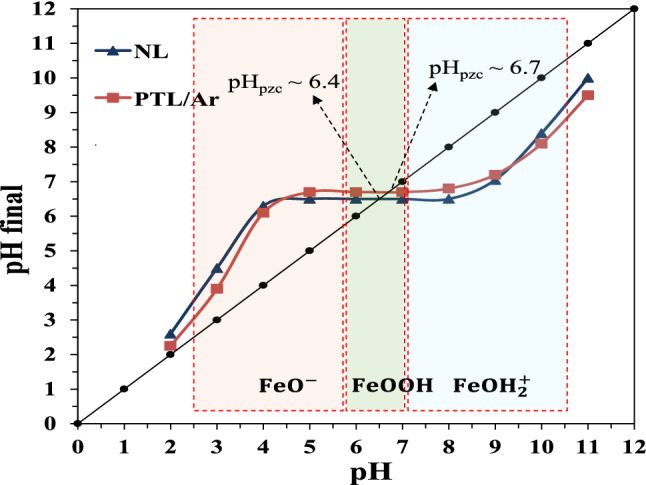
Determination of point of zero charge for NL and PTL/Ar.

The pH_PZC_ was determined by plotting the final pH versus the initial pH. As depicted in Fig. 8, the pH_PZC_ of the NL and PTL/Ar were measured 6.4 and 6.7, respectively, indicating successful hydroxylation of limonite after Ar-plasma treatment. The net surface charge of limonite is zero at pH_PZC_. Furthermore, the surface of limonite positively charged when pH_solution_ < pH_PZC_ and negatively charged when pH_solution_ > pH_PZC_^32^. According to Eq. (3), the protonation of limonite happened when pH of solution was lower than 6.4 and, deprotonation was happened when pH of solution was higher than 6.4, according to Eq. (4)^12^.

3 $$ {\text{FeOH}} + {\text{H}}^{ + } \leftrightarrow {\text{FeOH}}_{{2}}^{ + } \quad {\text{pH}}_{{{\text{solution}}}} < {\text{pH}}_{{{\text{PZC}}}} $$

4 $$ {\text{FeOH}} + {\text{OH}}^{ - } \leftrightarrow {\text{FeO}}^{ - } + {\text{H}}_{{2}} {\text{O}} {\text{pH}}_{{{\text{solution}}}} > {\text{pH}}_{{{\text{PZC}}}} $$

### Oxidation process

#### Effect of different oxidation processes

**Figure 9:**
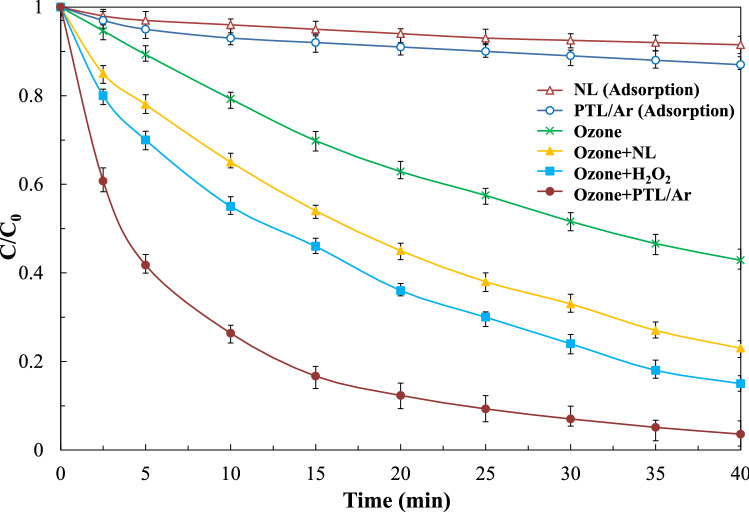
Comparison of PhP degradation efficiency with different oxidation processes ([PhP]_0_ = 30 mg/L, [O_3_]_0_ = 11 mg/L, catalyst dosage = 0.75 g/L, pH_0_ = 6.7).

The degradation of PhP was evaluated in the absence and presence of ozonation. Figure 9 shows the comparison of PhP degradation with different processes including adsorption with NL and PTL/Ar, ozonation, catalytic ozonation using NL and PTL/Ar. As can be seen from this figure, the adsorption of PhP was 12% on PTL/Ar and 8% on NL after 40 min of treatment. A little increase in PhP degradation through adsorption on PTL/Ar is attributed to the nanostructured limonite formation under plasma treatment which increased the specific surface area. In the oxidation processes, O_3_/PTL/Ar showed the most efficient process. The degradation of PhP reached to 58%, 77% and 97% through O_3_, O_3_ + NL and O_3_ + PTL/Ar, respectively. A sharp increase in PhP degradation in the O3 + PTL/Ar process indicated good capability of this process toward generation of reactive species. The catalytic performance of limonite is defined by two mechanisms: 1-presence of hydroxyl groups on the surface and, 2- presence of iron ions in the limonite structure^33^. The interaction of ozone and surface hydroxyl groups make a series of chain reactions which causes to formation of reactive species specially hydroxyl radicals. The proposed mechanism for ozone decomposition on PTL/Ar surface can be described as Eqs. (5)–(10)^34^.

5 $$ {\text{PTL}} - {\text{OH}} + {\text{O}}_{3} \leftrightarrow {\text{PTL}} - {\text{OH}}({\text{O}}_{3} )_{s} \leftrightarrow {\text{PTL}} - {\text{O}}{}^{ \cdot } + {\text{HO}}_{3} {}^{ \cdot } $$

6 $$ {\text{PTL}} - {\text{O}}{}^{ \cdot } + {\text{H}}_{2} {\text{O}} \to {\text{PTL}} - {\text{OH}} + {}^{ \cdot }{\text{OH}} $$

7 $$ {\text{HO}}_{3}^{ \cdot } \leftrightarrow {\text{H}}^{ + } + {}^{ \cdot }{\text{O}}_{3}^{ - } $$

8 $$ {\text{HO}}_{3}^{ \cdot } \to {}^{ \cdot }{\text{OH}} + {\text{O}}_{2} $$

Furthermore, plasma removes some impurities and more iron is available on the surface of limonite after plasma treatment^33^.

9 $$ {\text{Fe}}^{2 + } + {\text{O}}_{3} \to {\text{FeO}}^{2 + } + {\text{O}}_{2} $$

10 $$ {\text{FeO}}^{2 + } + {\text{H}}_{2} {\text{O}} \to {\text{Fe}}^{3 + } + {}^{ \cdot }{\text{OH}} + {\text{OH}}^{ - } $$

Moreover, the kinetic study of PhP degradation was studied. From Table 3 pseudo first order kinetics behavior of PhP degradation can be indicated for different processes. Adsorption on NL and PTL/Ar processes had the lowest apparent PhP degradation rate constants of 0.0026 and 0.0040, respectively. In contrast, the O_3_ + PTL/Ar process had the highest reaction rate constant due to synergistic effect between ozonation and adsorption processes. The synergic factor can be calculated from Eq. (11):

11 $$ {\text{synergy}}\,{\text{factor}} = \frac{{k^{\prime}{ }\,{\text{catalytic}}\,{\text{ozonation}}}}{{k^{\prime}{ }\,{\text{adsorption}} + k^{\prime}\,{\text{ozonation}}}} $$

**Table 3:** The apparent pseudo-first-order rate constants, corresponding linear regression coefficients and synergy factor of decay PhP by various processes on.

No	Process	k_app,PhP_ (min^−1^)	Correlation coefficient (R^2^)	Synergy factor	Reference
1	NL	0.0026	0.952	–	This study
2	PTL/Ar	0.0040	0.938	–	This study
3	O_3_	0.0219	0.998	–	This study
4	O_3_ + NL	0.0378	0.995	1.54	This study
5	O_3_ + H_2_O_2_	0.0488	0.988	–	This study
6	O_3_ + PTL/Ar	0.0936	0.959	3.71	This study
7	O3	0.072	0.980	–	^35^
8	TiO_2_/UV	0.026	0.990	–	^35^
9	O_3_ + TiO_2_/UV	0.084	0.970	2.11	^35^

The rate constants of adsorption by PTL/Ar and sole ozonation processes were 0.004 min^−1^ and 0.0219 min^-1^, respectively, while for O_3_ + PTL/Ar process was 0.0936 min^−1^. Consequently, the synergy factor value was calculated at 3.71 which mean significant enhancement was occurred through combination of processes. Fathinia et al.^35^ also reported synergy factor for photocatalytic ozonation of PhP by TiO_2_ coated on ceramic plates. As can be seen in Table 3 the highest synergy factor was reported about 2.11 for PhP degradation at the optimized operating condition which is lower than synergy factor of O_3_ + PTL/Ar process. This can be explained by higher ROSs generation in presence of PTL/Ar in ozonation process. As a result, O_3_ + PTL/Ar process was selected as the desired catalyst for further catalytic ozonation experiments.

#### Effect of initial PhP concentration

**Figure 10:**
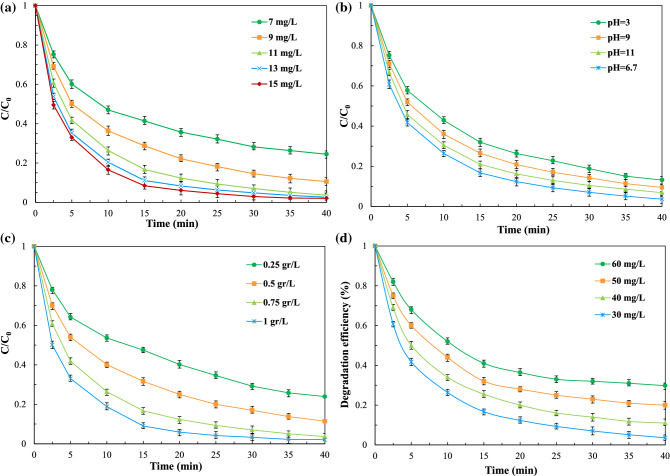
The influence of different parameters on the PhP degradation in the O_3_ + PTL/Ar system (**a**) ozone concentration ([PhP]_0_ = 30 mg/L, catalyst dosage = 0.75 g/L, pH_0_ = 6.7), (**b**) initial solution pH ([PhP]_0_ = 30 mg/L, [O_3_]_0_ = 11 mg/L, catalyst dosage = 0.75 g/L), (**c**) catalyst dosage ([PhP]_0_ = 30 mg/L, [O_3_]_0_ = 11 mg/L, pH_0_ = 6.7), (**d**) initial PhP concentration ([O_3_]_0_ = 11 mg/L, catalyst dosage = 0.75 g/L, pH_0_ = 6.7).

Figure 10a displayed the effect of initial contaminant concentration on the degradation of PhP in the O_3_ + PTL/Ar process. Experiments were performed at constant PTL/Ar dosage (0.75 g/L), ozone concentration (11 g/L), pH value (6.7) but at different initial PhP concentrations of 30, 40, 50 and 60 mg/L. The degradation of PhP was decreased from 98 to 71% by increasing its initial concentration from 30 to 60 mg/L, respectively. The degradation efficiency of PhP depends on the rate of radical generation. Whiles the oxidant concentration is constant, a competition occurred between the generated intermediates and byproducts with parent pollutant in reacting with radicals at high initial pollutant concentration^36^. Therefore, the possibility of ROSs reaction with PhP molecules decreases, which leads to reduction in degradation efficiency of oxidation process. A similar phenomena was observed by Khataee et al.^37^ that the degradation of textile dye in the sono-Fenton/pyrite process was decreased as initial dye concentration increased.

#### Effect of initial pH

The initial pH of solution is one of the significant parameters in the heterogeneous processes. It was stated in the literature that the oxidation process conducted mainly near or on the surface of catalyst due to the short life-time of free radicals. Therefore, it is desirable that the contaminant not repelled but attracted to the surface. Considering the pK_a_ of PhP (5.05) and pH_PZC_ of nanocatalysts (6.7), so in the acidic condition both PhP repelled from the surface. In such condition the surface charge density of the nanocatalyst become positive while the PhP molecules are in cationic form thus an electrostatic repulsion forces occurred. On the other hand, in the alkaline condition the negative charge density of surface and anionic form of PhP molecules hinder the contaminant adsorption and consequent reactions. In the moderate pH as Fig. 10b shows in the moderate pH value (6.7) the most effective PhP degradation was observed. In this pH value the surface of fabricated samples are negative and contrary the PhP molecules are in cationic form. Therefore, there is an attraction between contaminant molecules and nanocatalyst surface.

#### Effect of catalyst dosage

The influence of catalyst loading on the performance of oxidation process is demonstrated in Fig. 10c. As depicted, the degradation efficiency of PhP increased as the initial catalyst dosage ranged from 0.25 to 1 g/L due to the enhancement in active sites and accordingly, more radical were generated on the surface of nanocatalyst^9^. However, no significant difference was observed in the degradation efficiency of PhP between catalyst dosages of 0.75 and 1 g/L. Therefore, 0.75 g/L was selected as the optimum dosage of PTL/Ar nanocatalysts for further experiments. In the similar study conducted by Liu et al.^38^, no noticeable difference was observed for degradation of p-nitrophenol when limonite dosage changed from 2 to 4 g/L.

#### Effect of initial ozone concentration

The effect of initial ozone concentration was evaluated over the range of 7–13 mgO_3_/L at constant pH, PhP concentration and catalyst dosage of 6.7, 30 mg/L and 0.75 g/L, respectively and the results are presented in Fig. 10d. The results showed that the degradation efficiency raised from 77 to 99% as ozone concentration increased from 7 mg/L to 13 mg/L. A direct relation between pollutant degradation efficiency and initial ozone concentration is attributed to the production of more reactive species specially hydroxyl radicals^33^. It should be noted that the final PhP degradation was the same in the experiments containing 11, 13 and 15 mgO_3_/L. Therefore, 11 mg/L ozone concentration was selected as optimum value for further experiments.

### Stability and reusability

**Figure 11:**
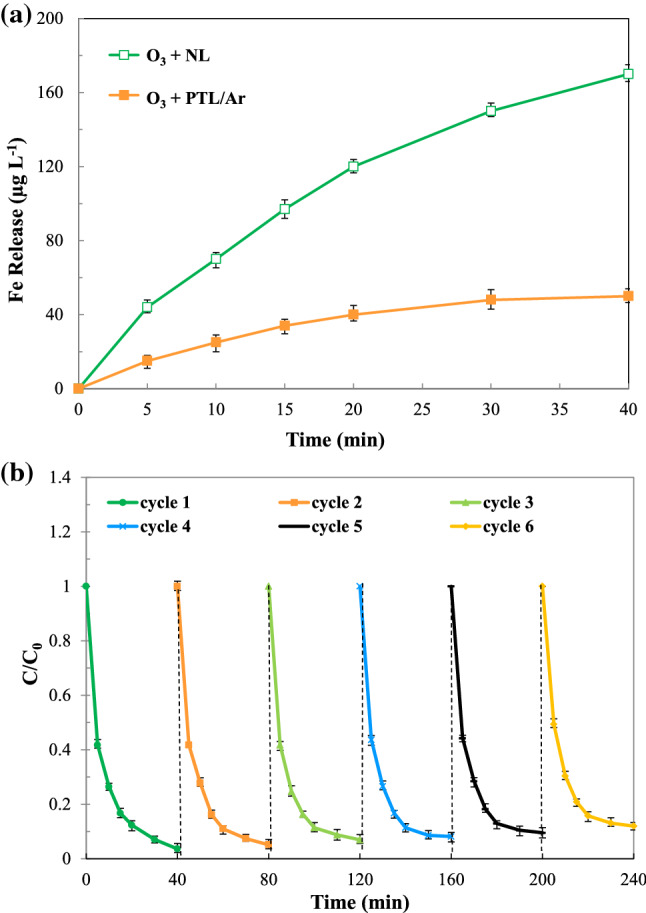
(**a**) Fe release in the O_3_ + NL and O_3_ + PTL/Ar processes (**b**) PhP degradation over 6 cycles in the O_3_ + PTL/Ar process ([PhP]_0_ = 30 mg/L, [O_3_]_0_ = 11 mg/L, catalyst dosage = 0.75 g/L, pH_0_ = 6.7).

The stability of catalyst plays an important role in the long-term operation. For this purpose, the Fe content released in the solution was analyzed before and after oxidation process by AAS analysis. The results of Fig. 11a showed that 0.170 mg/L and 0.048 mg/L Fe ions were detected in the O_3_ + NL and O_3_ + PTL/Ar processes, respectively. Therefore, the plasma treatment significantly decreased the amount of Fe release to the reaction medium. The improvement in catalyst stability after plasma treatment also was reported by researchers^24,37^. In order to investigate the reusability of the catalyst, the performance of PTL/Ar after 6 runs was evaluated. The results of reusability study were illustrated in Fig. 11b. The efficiency of PhP degradation decreased from 98% in the first run to 89% in 6th run, indicating no noticeable loss was observed in the PTL/Ar activity after 6 runs. The stability of catalyst might be because of the in-situ catalyst regeneration or repulsion of the detected by-products/reactant into the bulk^39^.

### TOC removal and electrical energy efficiency

**Figure 12:**
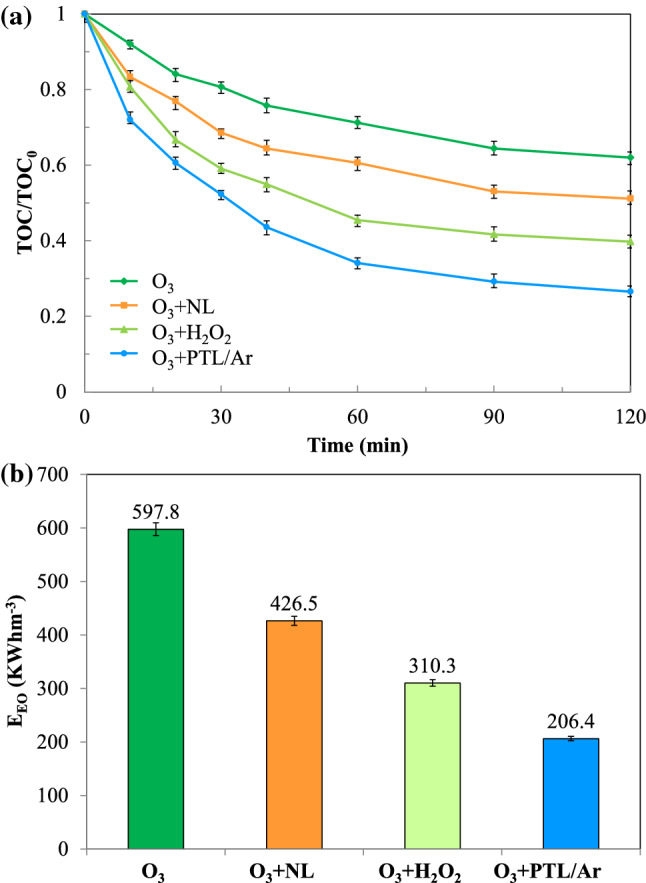
Comparison of TOC removal (a) and electrical energy consumption (b) in different oxidation processes, ([PhP]_0_ = 30 mg/L, [O_3_]_0_ = 11 mg/L, catalyst dosage = 0.75 g/L, [H_2_O_2_]_0_ = mg/L, pH_0_ = 6.7, P = 69 W for production of 4 g/h O_3_).

The mineralization of PhP oxidation process was evaluated by measuring total organic carbon (TOC). As illustrated in Fig. 12, different ozonation processes were applied including O_3_, O_3_ + NL, O_3_ + H_2_O_2_ and O_3_ + PTL/Ar. The results showed that 37, 50, 60 and 71% of the TOC was removed in the O_3_, O_3_ + NL, O_3_ + H_2_O_2_ and O_3_ + PTL/Ar processes, respectively. As can be seen the highest mineralization of PhP was observed in O_3_ + PTL/Ar process. Comparing the ozonation process in the presence of NL and PTL/Ar revealed that the plasma treatment of NL highly affect the mineralization of PhP.

In this research, the electrical energy per order (E_EO_) of the above processes was calculated using Eq. (12) as follows^40^:

12 $$ E_{EO} = { }\frac{{{\text{P }} \times {\text{ t }} \times { }1000}}{{V \times \log \left( {{ }\frac{{TOC_{0} }}{{TOC_{f} }}} \right)}} $$

where P is the input power (kW) of the system, t is the process time (h), V is the volume of aquatic solution (L), TOC_0_ (mg/L) and TOC_f_ (mg/L) are the initial and final total organic carbons, respectively. The E_EO_ values for degradation of PhP according to optimum condition were calculated and the results are shown in Fig. 12. However, it seems that the combined processes are not cost-effectiveness but according to Fig. 12a,b the O_3_ + PTL/Ar process had the lowest energy consumption as well as highest TOC removal compared to O_3_, O_3_ + NL and O_3_ + H_2_O_2_ processes. Mehrjouei and co-workers^11,41^ compared the economic aspects of photocatalytic oxidation, photo and catalytic ozonation processes for degradation of dichloroacetic acid and oxalic acid and reported that photocatalytic ozonation represented the highest cost-effectiveness between the investigated processes.

### Elucidating the possible reaction mechanism of PhP degradation in catalytic ozonation

#### Scavenging study

**Figure 13:**
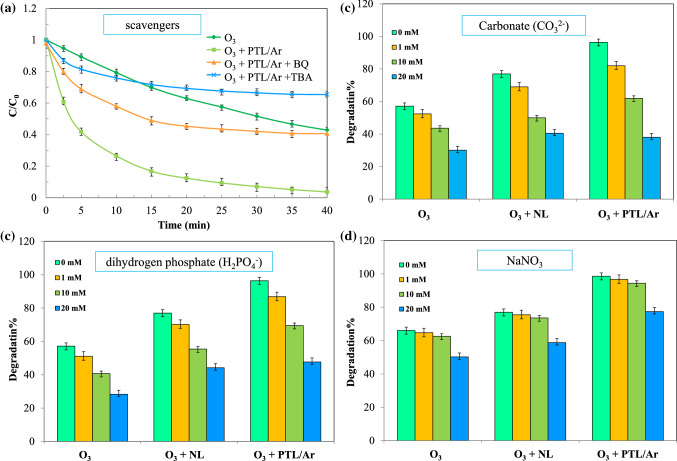
PhP degradation in the O_3_ + PTL/Ar system in the presence of different radical scavengers (**a**) and CO_3_^2−^ (**b**), H_2_PO (**c**) and NaNO_3_ (d) ([PhP]_0_ = 30 mg/L, [scavengers]_0_ = 10 mM, [O_3_]_0_ = 11 mg/L, catalyst dosage = 0.75 g/L, pH_0_ = 6.7).

Figure 13a,b show the effect of radical scavengers (TBA and BQ) and anionic ions (CO_3_^2−^, NO_3_^−^ and H_2_PO_4_^−^) on the performance of O_3_ + PTL/Ar process. TBA as free radical scavenger reacts with hydroxyl radical at a second-order-rate constant $(k_{{OH^{ \bullet } /TBA}}$ = (3.8–7.6) × 10^8^ 1/M.s). Also, BQ was used as superoxide radical scavenger. The concentrations of scavengers were too high in order to sufficiently quench the generated radicals instead of reacting with PhP. From Fig. 13a, almost complete PhP degradation was happen in the blank condition. In the experiment containing BQ, a decline in PhP degradation to 60% is noticed due to the high reactivity of TBA with hydroxyl radical. In the presence of TBA, strict scavenging was happened and, the degradation efficiency of PhP was reduced to 38%. The inhibitory effect of scavengers revealed that there are a few superoxide radicals in the system and, it was concluded that the hydroxyl radical was the main responsible for PhP degradation. The PhP degradation efficiency was decreased by addition of anionic salts to the solution medium (Fig. 13b,d). These anionic ions adsorbed on the PTL/Ar surface and occupied the surface active sites. Furthermore, the produced radicals can be consumed by the mentioned anions according to Eqs. (13)–(15)^9^:

13 $$ CO_{3}^{2 - } + {}^{ \cdot }OH \to CO_{3}^{ \cdot - } + OH^{ - } $$

14 $$ {\text{NO}}_{3}^{ - } + {}_{ }^{ \cdot } {\text{OH}} \to {\text{NO}}_{3}^{ \cdot } + {\text{ OH}}^{ - } $$

15 $$ {\text{H}}_{2} {\text{PO}}_{4}^{ - } + {}^{ \cdot }{\text{OH}} \to {\text{H}}_{2} {\text{PO}}_{4}^{ \cdot } + {\text{OH}}^{ - } $$

Therefore, the rate of radical production was decreased due to the presence of the salts and, carbonate had the greatest effect.

#### Dissolved ozone concentration and inorganic nitrogen evaluation

**Figure 14:**
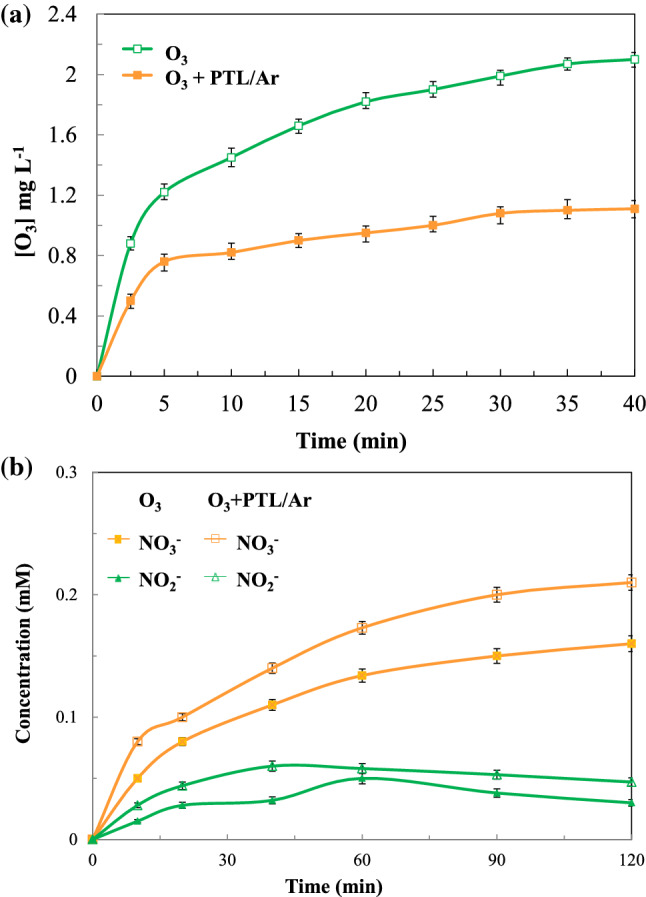
Dissolved ozone (**a**) and nitrogen evaluation (**b**) during ozonation and catalytic ozonation (PTL/Ar) processes.

The concentration of dissolved ozone was evaluated during ozonation and catalytic ozonation (with PTL/Ar) processes and shown in Fig. 14a. As can be seen in sole ozonation process the concentration of dissolved ozone was improved significantly and then reached to about 2 mg/L in 40 min. On the contrary in the catalytic ozonation with PTL/Ar lower dissolved ozone concentration was observed and after 30 min of process the changes were very negligible. The lower dissolved ozone concentration means higher portion of ozone molecules was decomposed. As a consequence of ozone decomposition the reactive oxidative species will be generated in the medium and hence the PhP degradation will be increased. The higher dissolved ozone concentration in the presence of Fe-based natural catalysts was also reported in previous works^12,42^. The release of inorganic ions (NO_3_^−^ and NO_2_^−^) from N heteroatoms as a consequence of PhP bond cleavage in sole and catalytic ozonation is presented in Fig. 14b. As it can be seen NO_3_^−^ and NO_2_^−^ ions were accumulated in the medium gradually from the beginning of processes. More nitrate ions were created in both processes compared to nitrite ions. Nitrite ion concentration increased at beginning of processes and followed by a gradual decrease which can be due to conversion to nitrate. Nitrate ions were formed in a more extent in comparison with nitrite and the concentration was increased over time of reaction. Furthermore, the amount of NO_3_^−^ and NO_2_^−^ ions release was higher in O_3_ + PTL/Ar process than sole O_3_. This means ozonation process in the presence of PTL/Ar nanocatalysts had more degradation efficiency compared to ozonation process alone. These findings are in consistence with the other researcher coutcomes^43^.

#### Analysis of PhP degradation intermediates and products

**Figure 15:**
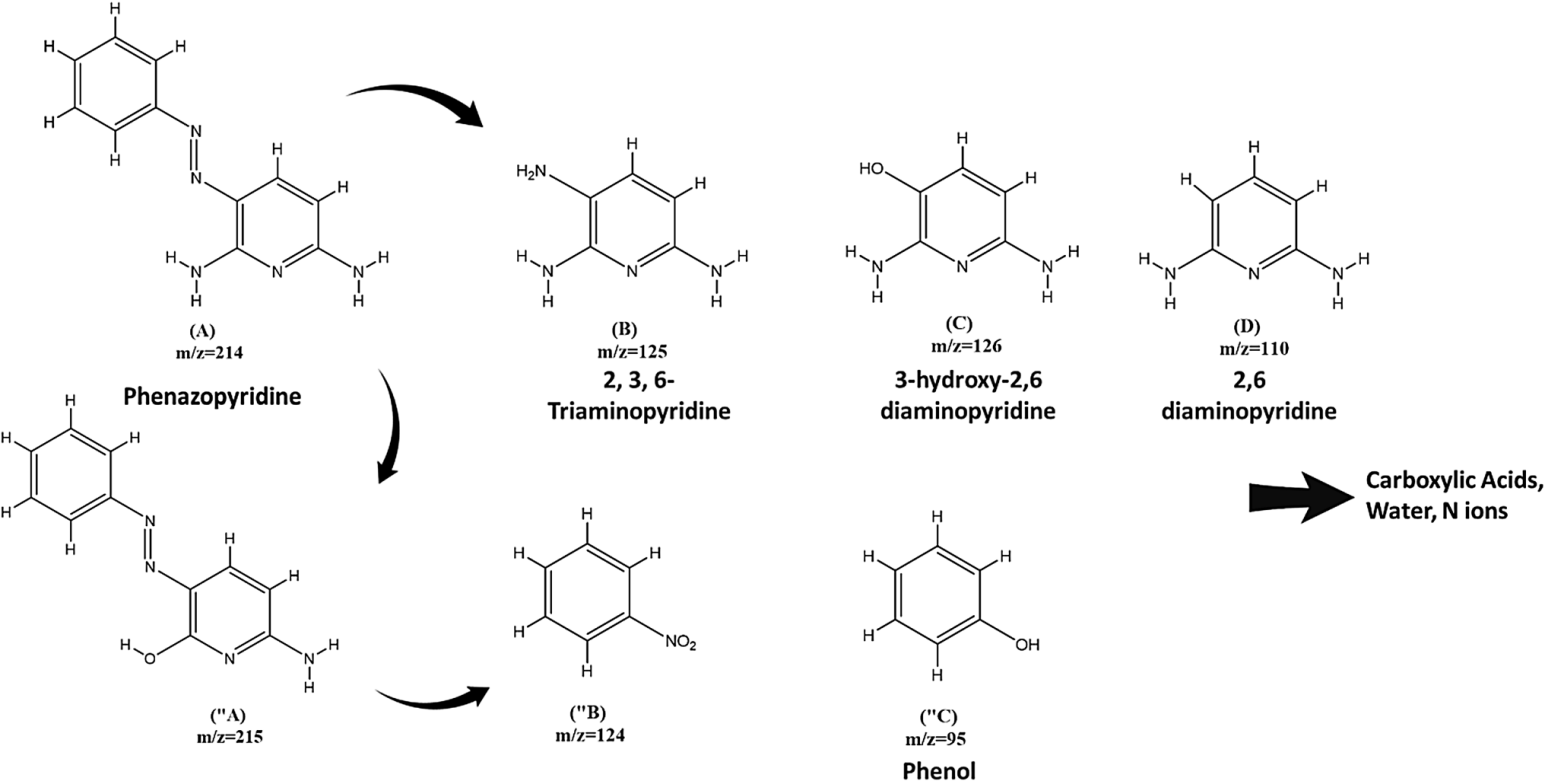
Proposed possible degradation pathways of PhP in catalytic ozonation processes using PTL/Ar.

The produced intermediates and by-products PhP degradation during catalytic ozonation by PTL/Ar was evaluated by LC–MS analysis. The detected oxidation intermediates are summarized in Fig. 15. According to the obtained results, the attack of hydroxyl radicals to the PhP molecule (m/z = 214) can produce intermediate with m/z = 125 (2,3,6-Triaminopyridine) as the result of N = N band cleavage. In addition the “A intermediate (m/z = 215) can be produced as a result of ^·^OH attack to N atom of amine group of PhP. 2,3,6-Triaminopyridine (A) degraded to 3-hydroxy-2,6-diaminopyridine with m/z of 126 which can furthered degraded to 2,6-diaminopyridine (m/z = 110). The intermediate with m/z of 124 (“B) was identified as a result of N=N bond cleavage of “A structure. Phenol compound (m/z = 95) was generated due to hydroxylation of “B bonds. Finally, multiple hydroxylation reactions and bond cleavage occurred and further reaction of ^·^OH and other ROSs with different structures led to production of end-products such as H_2_O, CO_2_, and N ions.

## Conclusions

In the present research, the beneficial limonite nanocatalysts were prepared by non-thermal glow discharge plasma under Ar atmosphere. Based on the characterization analyses, the small particle size, highly porous and rough nanostructure of plasma treated samples will improve the PhP degradation. A comparison between different degradation processes demonstrated that catalytic ozonation with PTL/Ar due to the higher specific surface area and total pore volume led to 96.4% degradation and 74% mineralization during 40 and 120 min of process, respectively. The excellent properties of nanostructures which were achieved by plasma treatment enhanced the penetration of pollutant molecules and thus improved the catalytic activity of PTL/Ar. The optimum amount of pH value, catalyst loading, initial ozone and contaminant concentration were observed to be 6.7, 0.75 m/L, 11 mg/L and 30 mg/L, respectively. Lower Fe ion release in addition to high reusability and stability during the catalytic ozonation process with PTL/Ar can be due to strengthening of the crystalline structure after plasma treatment in comparison with raw samples. The mineralization and electrical energy consumption results revealed that, in contrast to the O_3_, peroxone and O_3_ + NL processes, the O_3_ + PTL/Ar process expressed the highest mineralization and lowest electrical energy consumption under the optimized conditions. The PhP degradation mechanism was explored by different inorganic and organic scavengers as well as O_3_ concentration evaluation which proved that the indirect ozone attack was the dominant degradation pathway. The main intermediates during PhP oxidation by catalytic ozonation processes using PTL/Ar were identified using LC–MS technique. Considering the supreme activity and stability of the plasma treated nanocatalyst, it can be concluded that PTL/Ar has potential for further practical applications.
